# Subclinical diagnosis of cisplatin-induced ototoxicity with biomarkers

**DOI:** 10.1038/s41598-022-23034-x

**Published:** 2022-10-27

**Authors:** Charles Generotti, Brandon C. Cox, Jarnail Singh, Deborah Hamilton, Erica McKenzie, Bert W. O’Malley, Daqing Li

**Affiliations:** 1 Department of Otolaryngology–Head and Neck Surgery, Perelman School of Medicine, University of Pennsylvania, 421 Curie Blvd., BRB 1212, Philadelphia, PA 19104 USA; 2 Department of Pharmacology, Southern Illinois University School of Medicine, 801 N. Rutledge St, Springfield, IL 62702 USA; 3 Department of Civil and Environmental Engineering, Temple University, Philadelphia, PA 19122 USA; 4 University of Maryland School of Medicine, 655 W. Baltimore Street, Baltimore, MD 21201 USA

**Keywords:** Diagnostic markers, Predictive markers, Prognostic markers, Head and neck cancer, Disability

## Abstract

A mouse model with cisplatin-induced ototoxicity was used in addition to human samples from the ITMAT Biobank at the University of Pennsylvania. Mouse auditory brainstem responses (ABR), inner ear histology, perilymph cisplatin sampling, and measurement of serum prestin via ELISA were performed. Human serum prestin level was measured via ELISA in patients with otological issues after cisplatin treatment and compared to matched controls. Serum prestin was significantly elevated before ABR threshold shifts in mice exposed to cisplatin compared to control mice. Prestin concentration also correlated with the severity of hearing threshold shifts in mice. After an extended rest post-cisplatin treatment, prestin returned to baseline levels in mice and humans. Prestin was significantly elevated in the serum before the onset of objective hearing loss and correlated with the severity of hearing damage indicating that prestin may function as an effective biomarker of cisplatin-induced ototoxicity. Human serum prestin levels responded similarly to mice > 3 weeks from ototoxic exposure with decreased levels of prestin in the serum.

## Introduction

The relationship between ototoxicity and commonly used medicines and therapies has been known for over half a century, many of which result in permanent hearing damage^1^. One of the most common ototoxic medications used in clinical practice is cisplatin (CDDP). CDDP is widely used to treat cancers of the head and neck, lung, and genitourinary system and is included in the World Health Organization Model List of Essential Medicines, demonstrating its importance as a global chemotherapeutic agent^2^. Unfortunately, rates of CDDP-induced ototoxicity can be as high as 94%, with ototoxicity occurring in one-third of patients after receiving a single dose of CDDP^3^. The hearing loss typically appears within days, causing symmetrical, progressive, dose-dependent bilateral sensorineural hearing loss (SNHL) that is frequently accompanied by tinnitus and is worse at high frequencies^4,5^. In the pediatric population, cisplatin-induced hearing loss within the speech range can have a profound impact on cognitive development, leading to life-long complications^6–8^.

CDDP-induced ototoxicity leads to cessation or modification of the cancer treatment plan, negatively impacting patient care. While patients undergoing therapy are routinely screened for hearing loss, abnormal results likely signify irreversible hearing damage beyond feasible intervention^9^. Prophylactic strategies or early treatment are of paramount importance in avoiding changes in optimized treatment regimens and reducing the disease burden caused by ototoxic drugs. Responding to damage in a timely fashion is dependent on the identification of a subclinical diagnosis of cisplatin-induced ototoxicity. We believe the subclinical diagnosis can be established using a reliable early biomarker for ototoxicity.

Several potential biomarkers of ototoxicity are well known in the literature through animal models, yet none have been fully established for use in humans. The most promising thus far is prestin, a motor protein essential for hearing function that is located in the outer hair cells of the inner ear and whose expression is conserved across species^10^. Prestin has been found to be acutely elevated in the serum after a variety of ototoxic insults in previously published literature. Cisplatin, aminoglycosides, and acoustic trauma have all led to prestin elevation in serum via ELISA in mice, guinea pigs, and recently in humans with Sudden Sensorineural Hearing Loss^10–15^. Prestin elevation has been shown to occur up to one day before the onset of clinical diagnosis of hearing loss, possibly presenting a therapeutic window for future treatment^11,14,15^. Prestin has also been shown to return to pre-exposure levels after removal of the ototoxic agent^12^.

In this study, we assess the relationship of ototoxic damage, serum prestin concentration, CDDP perilymph concentration, and inner ear morphology in the setting of cisplatin-induced ototoxicity in a clinically relevant mouse model and in human serum samples from patients treated with cisplatin provided through the Penn Medicine BioBank. Our primary objective is to determine if serum prestin can be used to make the subclinical diagnosis of cisplatin-induced ototoxicity and monitor the severity of hearing damage. We would also like to assess if prestin responds predictably in humans compared to our animal model. We hypothesize that when the inner ear structures are challenged by cisplatin, prestin will increase significantly in the serum in a sequential and predictable fashion in both the mice and in humans before the development of symptomatic and audiologically determined hearing loss and a clinical diagnosis of ototoxicity.

## Methods

### Animal study design

This study was approved by the Institutional Animal Care and Use Committee (IACUC) at the University of Pennsylvania and all methods were performed in accordance with the relevant guidelines and regulations. Six-week-old CBA/J mice from The Jackson Laboratories (Bar Harbor, ME) were weighed prior to procedures and kept in standard housing with free access to food and water. Each day procedures took place, mice were observed closely for signs of weight loss, isolation, poor social interaction, and general distress.

Mice were divided into eleven groups of six mice with an additional group of eight mice for control. All mice had baseline tone-evoked Auditory Brainstem Responses (ABR) performed before cisplatin exposure. The 11 groups of mice were treated with three cycles of cisplatin at 3.5 mg/kg via intraperitoneal injections over four days with a 10-day rest period in between cycles using a protocol adapted from Fernandez et al. seen in diagram 1^16^. Cisplatin was diluted 1:1 with 0.9% saline for hydration. A single group of six mice was sacrificed at recovery days 1, 5, and 10 after each cycle of cisplatin, with the two remaining groups, sacrificed at day three during the second cycle of cisplatin administration and 14 days after the end of the final recovery period. The additional group within cycle two of cisplatin administration was added to the study based on preliminary data demonstrating this timepoint as the first in which mice developed significant hearing loss. Serum collection, ABRs, perilymph sampling, and cochlear tissue collection were performed at the time of sacrifice.

#### Serum prestin ELISA

Roughly 0.5–1.0 ml of blood was collected via cardiac puncture under ketamine/xylazine and allowed to clot for 30 min before centrifugation for 15 min at 3000 rpm. Serum samples were stored at – 80 °C until prestin concentration was measured using a cavy SLC26A5 ELISA kit (MyBioSource.com) as described in the manufacture’s manual. Optical density was measured at 450 nm using a Spectramax 190 reader (Molecular Devices), and data were compiled with the SoftMax Pro6 software package.

#### Auditory brainstem responses

Anesthetized CBA/J mice were placed on a heated pad in a soundproof chamber. ABRs were recorded in response to clicks and tones (4, 8, 16, 24, and 32 kHz) between 20 and 100 dB in 5 dB increments using an RZ6 multi I/D signal processor and BioSigRZ software (TDT, Alachua FL). ABRs were performed at baseline, before cisplatin exposure and before euthanasia at all time points. Animals with evidence of hearing loss at baseline were not included in the study.

#### Perilymph sampling and tissue morphology

Mice were euthanized by cervical dislocation while sedated, and the temporal bones were immediately dissected from the skull base. Using a glass tipped micro-pipette, roughly 0.3µL of perilymph was extracted from the oval window. Perilymph was immediately placed in 10 µl PBS solution and stored at 4 °C until testing using ICP-MS. The cochleae were flushed and submerged in 4% PFA and left overnight at room temperature. Temporal bones were removed, placed into 1% PBS, and stored at 4 °C. Whole mount dissection of the decalcified cochleae and immunostaining was performed as previously described where the cochlea was divided into three turns (apex, middle and base) that were approximately equal in length. Primary antibodies included myosin VIIa (1:200; cat# 25-6790; Proteus Biosciences) and prestin (1:200; cat# sc-22692; Santa Cruz Biotechnologies)^17^. Alexa conjugated secondary antibodies were used at 1:1000 dilution and Hoechst was used at 1:2000. Confocal images were taken using a Zeiss LSM800 microscope and images were processed using Zen blue software. Hair cells were manually counted in two, 150 µm regions per cochlear turn. Mice groups being assessed were blinded to technician performing hair cell counts.

#### Human case–control design

Using the ITMAT biobank through the University of Pennsylvania, we queried a database of over 60,000 subject samples stored at − 80 °C. Our initial query included only subjects that had given samples to the biobank within 90 days of receiving cisplatin chemotherapy. Of these 77 subjects, 9 met criteria after reviewing each patient’s medical records for documented otological issues, including tinnitus, hearing loss, ear fullness, or vertigo. Vulnerable populations and patients with prior hearing loss, prior exposure to ototoxic agents, family history of otologic disorders, or inadequate samples available were excluded from the study. Cases were paired with a single age and gender-matched control without chemotherapy exposure or documented otological issues from the biobank. The average age of cases was 51.8 years old, with seven males and one female. Cases had varying degrees of cisplatin exposure with the addition of etoposide and gemcitabine in some cases. Serum samples were tested for prestin using a human SLC26A5 ELISA kit (mybiosource.com) in our lab and compared between the two groups, with one sample discarded since the measurement was below the detection limit.

### Data analysis

**Figure 1:**
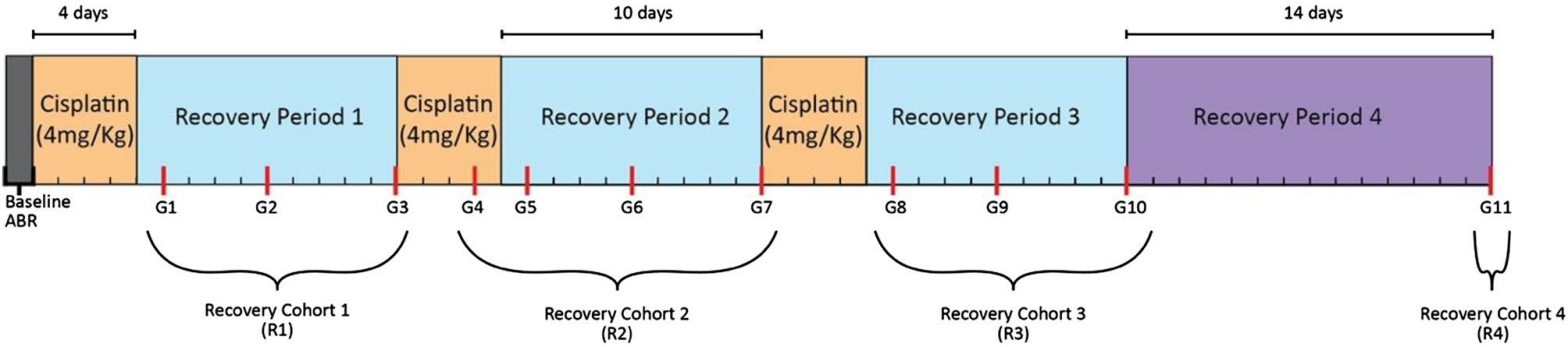
Timeline of experiment with outline of recovery cohorts. Red hashmarks represent sacrifice timepoints.

Mouse ABR, cisplatin perilymph samples, and cochlear morphological data were from the right ear of each mouse. ABR threshold shifts were calculated by subtracting baseline ABR thresholds from ABR threshold obtained at the time of sacrifice. Normality of distribution was tested using the Shapiro–Wilk test for all data. Statistical comparisons for mouse data were completed between each group using a Welch’s *t*-test with *P* set at 0.05 using Graphpad. Mouse data were treated as independent and human data was treated as matched pairs. Statistical comparisons were made by combining groups of mice after each recovery period to form recovery cohorts (R1-R4). The R1 cohort is a combination of the three groups of mice treated after the first cycle of cisplatin. R2 is the four groups of mice during and after the second cycle of cisplatin. R3 is the combination of the three groups after the third cycle of cisplatin, and R4 is the final group of mice after an additional 14 days beyond the final rest period (Fig. 1). Human data were compared using Wilcoxon signed rank test. Case–control matching was completed using the ITMAT biobank database and medical record review for absence otologic signs and symptoms in history.

Eight mice in total were not included in the statistical analysis due to premature death related to cisplatin toxicity (1), experimental dilution error (1), premature euthanasia due to injury (2), insufficient serum sample (4).

### Ethics approval

No human subjects were directly recruited into this present study. All samples were from subjects of which informed consented was obtained voluntarily to participate in the collection of samples through the ITMAT biobank at the University of Pennsylvania and approved by the Penn Institution Review Board (IRB#813913 & IRB#817977) and is in accordance with all relevant guidelines and regulations. The animal study was approved by the IACUCU of the University of Pennsylvania (protocol #: 804352) and is in accordance with the relevant guidelines and regulations as well as the ARRIVE guidelines.

## Results

### ABR

**Figure 2:**
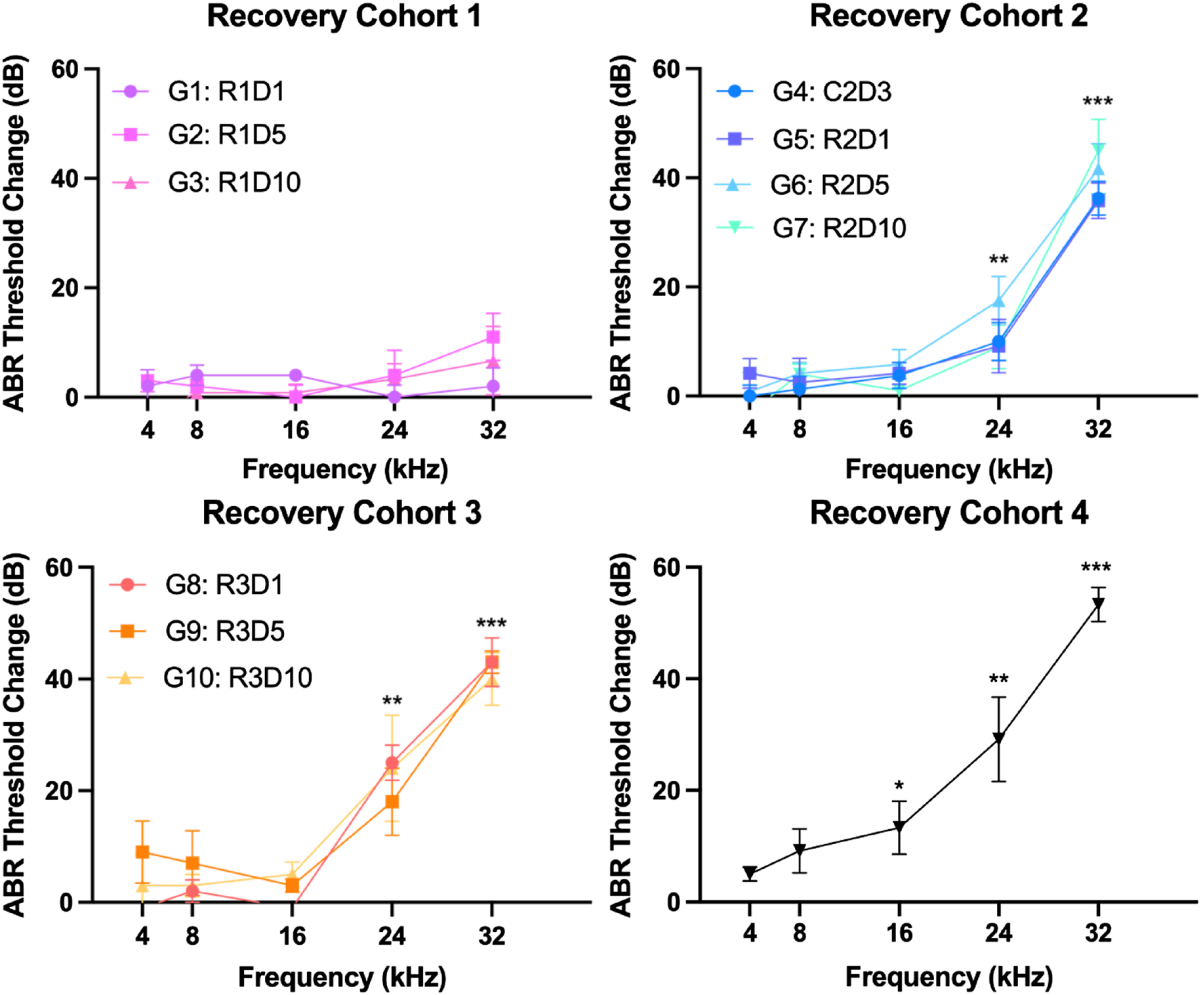
ABR threshold differences at time of sacrifice. Error bars represent standard error of the mean (SEM). Asterisks represent significance against control, * < 0.05, ** < 0.01, *** < 0.001.

After the first cycle of cisplatin, there were no significant changes in ABR thresholds compared to control at any frequency for R1 (n = 16). The first significant ABR threshold shift compared to control occurred after cycle 2 of cisplatin in R2 (n = 21) at 24 kHz, 12.86 ± 2.88 dB (mean ± SEM) (*p* = 0.00024) and at 32 kHz (40 ± 2.13 dB; *p* < 0.000001). R3 (n = 15) and R4 (n = 6) maintained significant ABR threshold shifts compared to control at 24 kHz and 32 kHz. R3 and R4 measurements below are shown as mean difference compared to control: at 24 kHz (R3 = 22 ± 4.14 dB; *p* = 0.000036 and R4 = 29.17 ± 3.74 dB; *p* = 0.0109) and at 32 kHz (R3 = 42 ± 2.12 dB; *p* < 0.000001 and R4 = 53.33 ± 3.07 dB; *p* < 0.000001). Additionally, R4 had a significant shift at 16 kHz of 13.33 ± 5.11 dB (*p* = 0.038) compared to control (Fig. 2).

**Figure 3:**
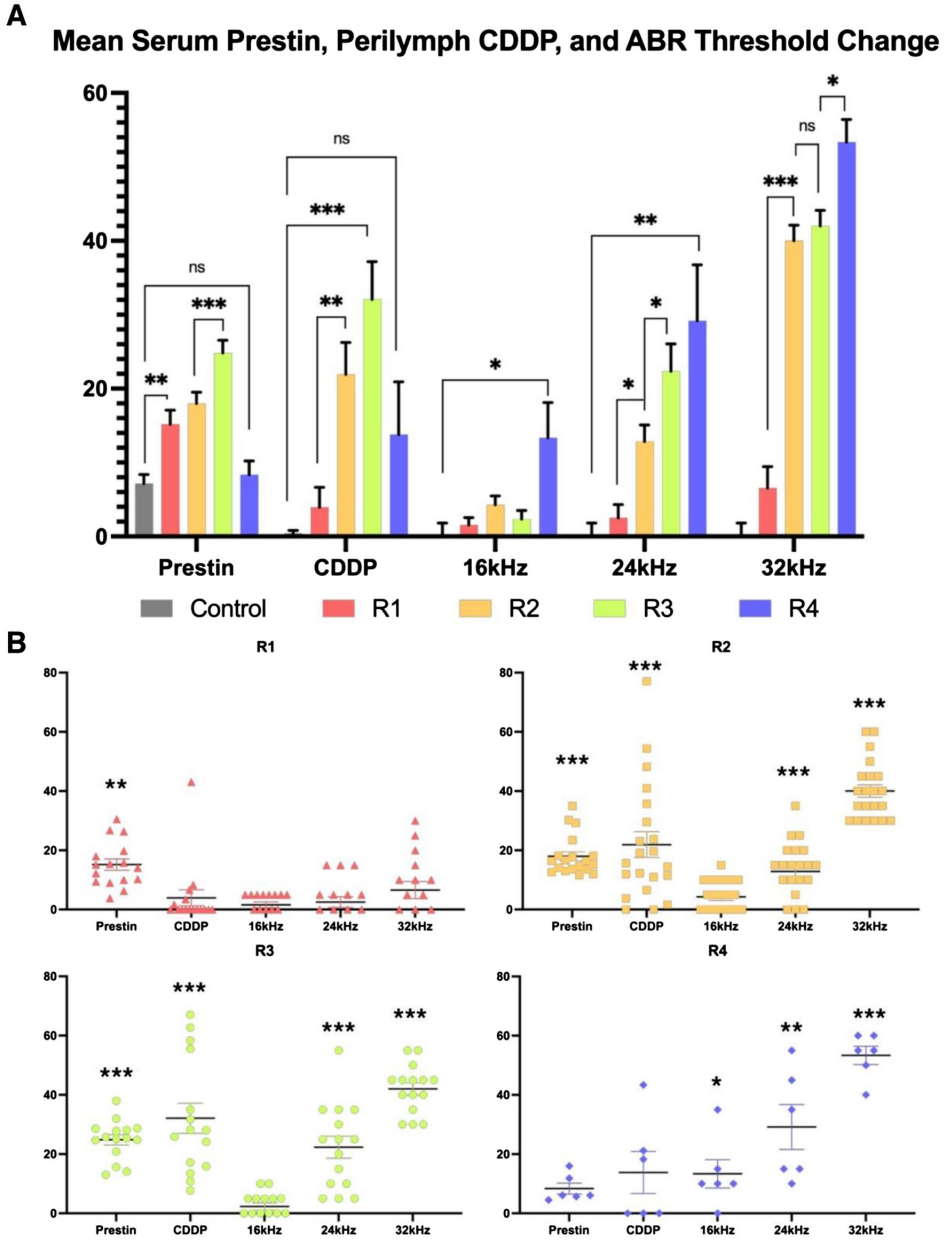
Serum prestin (ng/mL), cisplatin perilymph concentration (pg/mL), and ABR threshold shifts (dB) plotted together for each recovery group with comparisons shown between each group (**A**), and distributions for each recovery group plotted and compared against control (**B**). Error bars represent standard error of mean (SEM), * < 0.05, ** < 0.01, *** < 0.001.

After the second cycle of cisplatin, R2 had significantly elevated thresholds compared to R1 at 24 kHz, with a mean difference of 10.36 ± 3.59 dB (*p* = 0.00098), and at 32 kHz (33.44 ± 3.578 dB; *p* < 0.000001). After the third cycle of cisplatin, R3 had a significant threshold change at 24 kHz compared to R2 with a mean difference of 9.476 ± 4.327 dB (*p* = 0.0386). There was no significant difference at 32 kHz between R2 and R3. R4 had the worst hearing of all groups with a significant threshold shift compared to R3 at 32 kHz (11.33 ± 3.731 dB; *p* = 0.0124) (Fig. 3).

### Serum prestin concentrations

Serum prestin levels were significantly elevated prior to the onset of cisplatin-induced hearing loss in R1 compared to control, 8.049 ± 2.271 ng/mL (mean difference ± SEM) (*p* = 0.00182). There was no significant difference between mean R1 (15.18 ± 1.89 ng/mL) and R2 (17.96 ± 1.54 ng/mL) prestin levels. After completing cycle 3, R3 (24.83 ± 2.56 ng/mL) had significantly elevated mean prestin levels compared to both R1 and R2 with a 6.86 ± 2.31 ng/mL mean difference compared to R2 (*p* = 0.00586) (Fig. 3).

**Figure 4:**
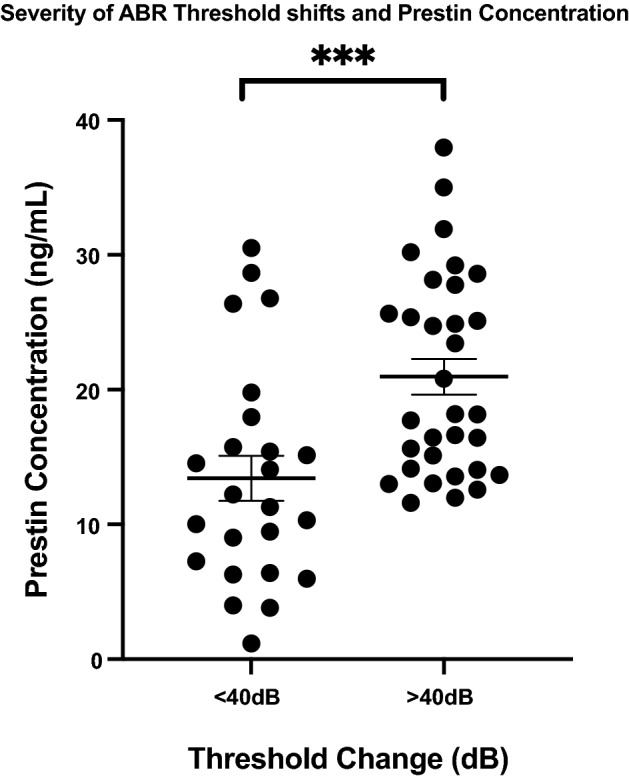
Serum prestin concentration (ng/mL) in mice with summative ABR threshold shifts of > 40 dB at 24 kHz and 32 kHz frequencies. Error bars represent standard error of mean (SEM), *** < 0.001.

In mice with hearing threshold shifts of more than 40 dB at 24 kHz and 32 kHz combined or > 35db at 32 kHz alone, serum prestin levels were significantly elevated in the serum when compared to mice with thresholds below these levels. The difference between the means was 7.535 ± 2.13 ng/mL (*p* = 0.000901) (Fig. 4). After 24 days post-cisplatin exposure, R4 had a significantly lower mean prestin serum concentration compared to all recovery cohorts with no significant difference compared to control (Fig. 3).

### Cisplatin perilymph concentration

Cisplatin perilymph concentrations increased alongside ABR threshold shifts and prestin concentration in serum (Fig. 3). CDDP levels were significantly elevated in R2 (21.91 ± 4.35 pg/mL, *p* = 0.000074) and R3 (32.09 ± 5.09 pg/mL, *p* = 0.000021) compared to R1 (3.95 ± 3.54 pg/mL) and control (0.41 pg/mL). R4 levels (13.8 ± 7.13 pg/mL) were not significant when compared to control (*p* = 0.118). R1 levels were equivalent to control.

### Hair cell morphology

**Figure 5:**
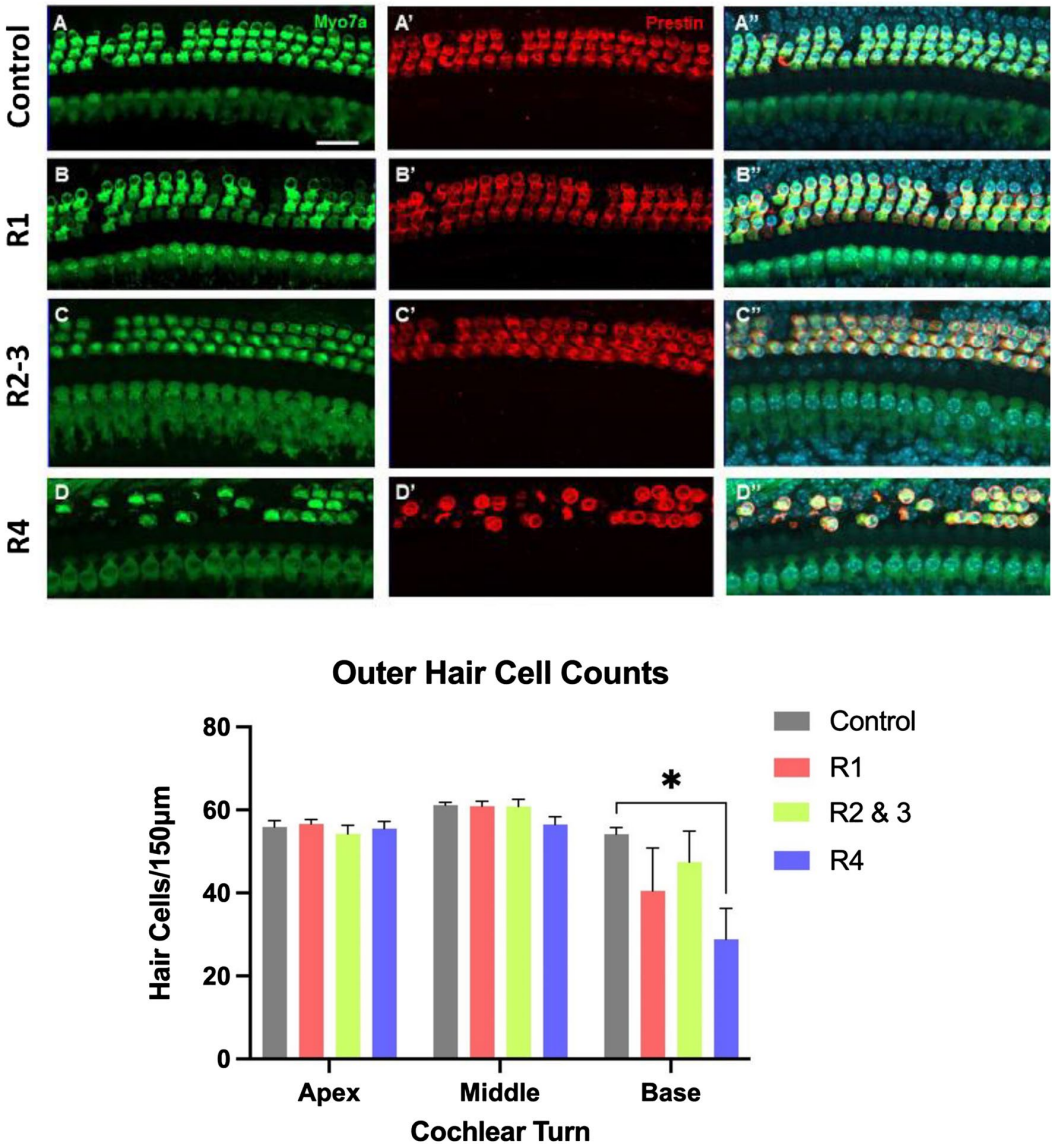
Representative confocal maximum projection images from the basal turn of control (**A**–**A**”) n = 6, R1 (**B**–**B**”) n = 4, R2-3 (**C**–**C**”) n = 5, and R4 (**D**–**D**”) n = 5 cochleae. Inner and outer hair cells are labeled by myosin VIIa (Myo7a, green) and outer hair cells are labeled by prestin (red). Nuclei are labeled by Hoechst (blue). Scale bar = 20 um. Quantification of outer hair cells in a 150 µm region per cochlear turn. Error bars represent standard error of mean (SEM), * < 0.05.

Four to five mice from each recovery cohort were selected for histology along with six control mice for a total of 20 mice. Outer and inner hair cells were counted using myosin VIIA staining and compared to control. There were no significant differences in the number of inner hair cells between groups. For outer hair cells, only R4 showed a significant decrease in the basal turn of the cochlea. Mean control numbers of outer hair cells in the basal turn were 54.17 ± 1.60 versus 28.80 ± 7.53 in R4 (mean ± SEM) (*p* = 0.026) (Fig. 5). All outer hair cells present in each sample expressed prestin in addition to myosin VIIa. There were no obvious changes in prestin expression pattern in any of the groups compared to control samples, but we did not quantify fluorescent intensity due to the limitations of this method (Fig. 5).

### Human prestin

**Figure 6:**
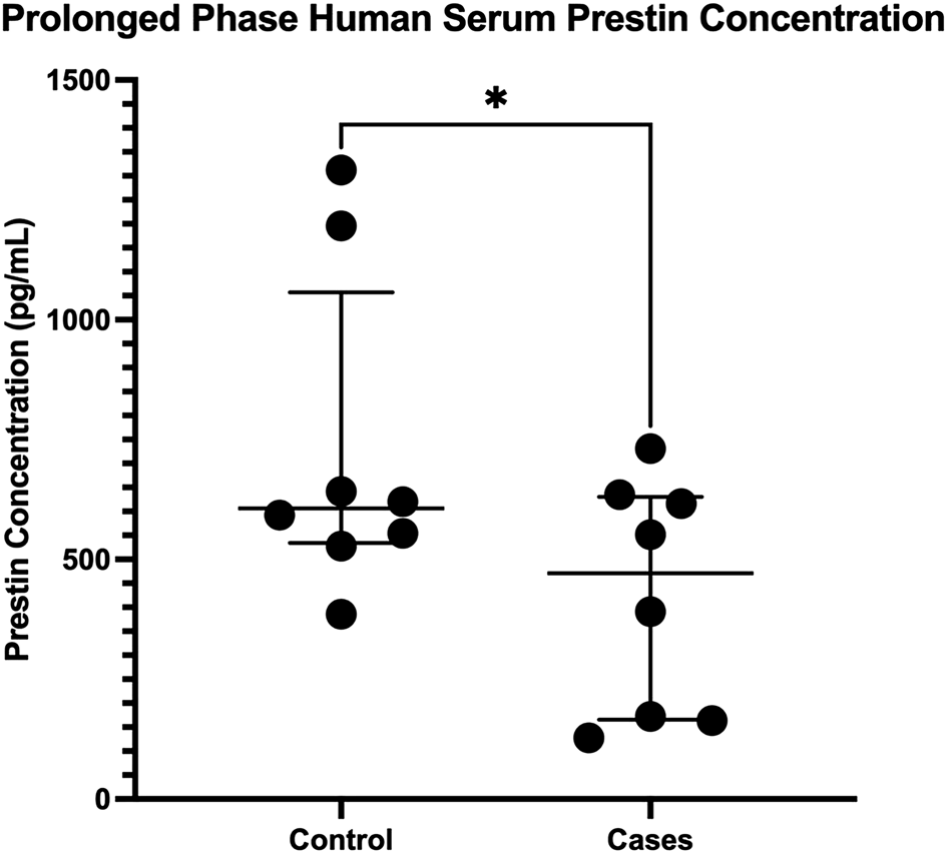
Prestin serum concentration in patients with signs and symptoms of hearing damage after receiving cisplatin compared to matched controls, median of differences = 328.5 pg/mL, *p* = 0.0156.

Patients exposed to cisplatin with a history of ontological symptoms or signs in their chart had significantly lower levels of prestin in their serum as seen in Fig. 6 (Median of differences = 328.5, *p* = 0.0156). The mean and SEM was 728.8 ± 118.4 pg/mL for control and 423.4 ± 85.78 pg/mL cases. The average time lapsed from cisplatin exposure to serum sampling was 47.25 days.

## Discussion

A successful biomarker for cisplatin-induced ototoxicity is capable of screening, diagnosing, and monitoring disease progression^18^. In this study, prestin was significantly elevated in the serum of R1 mice prior to the onset of significant ABR threshold shifts and OHC loss, demonstrating its ability to screen for and make the subclinical diagnosis of cisplatin-induced ototoxicity. These findings are consistent with previous reports in the literature showing an elevation of prestin in the serum before hearing changes occur^12,15^.

In our study, prestin was also found to correlate with the severity of ABR threshold shifts. Prestin levels increased significantly in the serum as ABR threshold shifts elevated with each cycle of CDDP (Fig. 4). This finding demonstrates that prestin holds the potential to monitor disease progression in the acute phases of inner ear stress. In 2016, Parham and Dyhrfjeld-Johnsen also showed similar findings with a positive linear relationship of serum prestin concentration and ABR threshold shifts^11^. Another study showed that prestin levels increased in the serum in response to a high dose injection of cisplatin versus a low dose. However, in this study, the low dose of CDDP may not have reached a threshold which challenged the inner ear significantly in their animal model^19^.

By measuring the concentration of CDDP in the perilymph, we found that after each cycle of systemic CDDP, there was a stepwise increase of CDDP in the perilymph of the mice. Mice with higher levels of CDDP in their perilymph had increased ABR threshold shifts and higher levels of prestin in their serum (Fig. 3). We believe the elevated levels of CDDP in the inner ear lead to additional ototoxic damage and inner ear stress that leads to increased gene expression and release of prestin in the serum. However, the etiology of prestin release from the OHC is beyond the scope of this study. In 2017, Breglio et al. also found CDDP to increase in a stepwise fashion in the cochlea of mice after each systemic dose of CDDP using a similar clinically relevant CDDP mouse model^20^. However, in their study, they measured CDDP throughout the entirety of the cochlea and found CDDP to be retained indefinitely within the stria vascularis, organ of Corti, and spiral ganglion with no significant change after 60 days of rest^20^. In our study, we found that within 24 days of CDDP exposure, R4 mice had CDDP levels return to non-significant levels in the perilymph. While R4 levels were still slightly elevated and almost significant compared to control, we believe the drop in CDDP concentration is likely due to the permeability differences between the endolymph and perilymph of the inner ear, and CDDP is likely still sequestered in other locations of the cochlear tissue^21^. This would also explain why the R4 group had hearing threshold shifts and morphology changes that continued to worsen compared to R3, suggesting that the accumulation of CDDP led to additional damage and outer hair cell loss over time. However, the potential efflux or redistribution of CDDP within the inner ear during this rest period may also play a role in the decline of prestin expression. Our results show that as CDDP levels dropped in the perilymph, prestin concentration in the serum returned to baseline.

Taken together from our current study and others, the data suggest that, following systemic administration, CDDP may enter the perilymph fluid before being sequestered by other inner ear structures causing ototoxicity. The inner ear CDDP entering mechanism may provide an important approach for blocking inner ear ototoxicity via perilymph dialysis.

Our study is the first to capture the relationship of prestin to sequential ototoxic exposures of CDDP, mimicking human dose protocols. Previous studies used a single high dose bolus of CDDP to generate hearing damage without additional exposure after prestin returned to baseline^11,12,15^. Our findings suggest that acutely elevated serum prestin is likely due to increased prestin expression in response to cisplatin-induced cellular stress rather than apoptosis of outer hair cells alone. This is supported by the insignificant changes to outer hair cell counts in samples with elevated levels of prestin in the serum (Fig. 5). While we did not observe any obvious changes in prestin expression in our histological analysis, we did not quantify fluorescent intensity due to the limitations of this method and thus small changes in prestin expression would not be detected. Most importantly, apoptosis of outer hair cells alone would likely cause a single spike in prestin with a return to baseline levels after the cells have spilled their contents. Prior literature supports this concept as well by showing that in response to noise-induced hearing loss, prestin was upregulated by 32–58% before returning to baseline at 4 weeks post-noise with outer hair cells intact^22,23^. In another study, prestin mRNA and protein expression increased threefold in response to two weeks of salicylate exposure (a known ototoxic agent) and returned to baseline 4 weeks after cessation without permanent hearing loss^24^.

R4 prestin concentration returned to baseline, and ABR threshold shifts remained elevated 24 days after completing cycle three of cisplatin. This finding is also consistent with prior studies, which showed prestin concentrations returning to baseline or below baseline after 14 days from cisplatin exposure^11,12,15^. One theory suggests that prestin levels below baseline may be related to a lower number of surviving outer hair cells which would be consistent with our histology data for R4^12^.

In our human analysis, we saw significantly lower prestin levels in our cases compared to controls. With an average duration between cisplatin exposure and sample collection of 47.25 days, our human cohort is comparable to R4. Both R4 and the human cases are beyond the 14-day window seen in the literature showing prestin returning to baseline, likely due to a decrease in prestin protein expression as the ototoxic stressor is removed. Lower levels of prestin in our human case samples compared to control correlates with our animal model and prior literature^11,12,15^. This response demonstrates that human prestin responds in a predictable fashion compared to the animal model in our study and in the literature. Another recent study, showed the human baseline levels of prestin negatively correlate with noise exposure, which is in line with our current data showing previous insult to the inner ear leads to lower baseline levels prestin^25^. Of note, our human control prestin concentrations are also within the ranges established by two studies measuring prestin in human serum: a recent publication on the reliability of serum prestin measurements in humans and a study on idiopathic sensorineural hearing loss^13,26^.

In our study we did not measure prestin levels from patients during their acute exposure to CDDP that would likely lead to elevated, rather than depressed levels of prestin in the serum. However, a recent study by Jalali et al., 2021 assessed serum prestin concentrations in 52 patients actively undergoing cisplatin-based chemotherapy. Similar to the acute findings in our animal model, they found early elevated prestin levels in the serum of patients exposed to cisplatin, and prestin levels were also elevated in patients with significant hearing damage^27^.

There are several limitations in both the animal and human portions of the present study. For our human samples from the ITMAT biobank, there was a low sample size of 8, and some patients were treated with additional chemotherapy agents such as gemcitabine and etoposide. While these drugs are not known for ototoxicity, we cannot be certain of their effect when given in combination with cisplatin^4,28^. Additionally, the classification of otologic symptoms from the chart is challenging and subjective from a timeline and severity standpoint. Without quantifiable hearing data for these patients, we are limited in the conclusions we can draw from this portion of the study. We also did not have baseline prestin levels for each individual patient, which is another major limitation as there is a fair amount of variability in baseline levels that correlate with environmental factors such as noise exposure^25^. Another limitation in comparing the human samples to our animal model is the discrepancy in cisplatin dosage. The mouse model in this study uses significantly higher doses to achieve consistent ABR threshold shifts, while humans typically receive much lower doses. The generalizability of our human study may be limited by the mostly male gender, older age, and small sample size available for testing, however, at baseline levels there were no significant differences in human serum based on gender or age^25^.

Another limitation of our study is the cross-sectional design used to collect serum for testing in our animal model. Ideally, it would be best to measure serum prestin concentration before and after cisplatin exposure in the same organism over time. However, this is not possible in mice due to their small blood volume. This was controlled for by using a sample size of six at each timepoint and comparing the averages for each group. Additionally, because there were no statistically significant changes between individual groups after each cycle of cisplatin, we were able to combine groups for more robust comparisons. Unfortunately, the R4 group had only six mice. A larger sample size during this time point would have helped determine the significance of the perilymph cisplatin concentration as it appears elevated but the data are not significant. It would have also been useful to assess for CDDP in other inner ear structures to compare to previous literature and to expand on the blood labyrinth barrier permeability characteristics. Based on the current study, we are planning to conduct a multi-institutional human clinical study to investigate several biomarkers, including prestin, which may be used for pre-clinical diagnosis for cisplatin-induced ototoxicity.

## Conclusion

This study demonstrates that prestin levels in serum can be used to make the subclinical diagnosis of cisplatin-induced ototoxicity prior to the onset of hearing threshold shifts. Prestin was also found to correlate with the severity of ototoxicity in our animal model. In our human study, prestin produced a predictable and similar response to our animal model which is consistent with the literature. Measurement of prestin in serum shows great promise as a pre-clinical biomarker for use to determine ototoxicity. Additional studies are needed to assess the relationship of prestin and cisplatin-induced ototoxicity in the acute setting.

## Data Availability

The datasets used and/or analyzed during the current study are available from the corresponding author on reasonable request.

## Abbreviations

ABR: Auditory brainstem response
CDDP: Cisplatin
